# Guominkang formula alleviate inflammation in eosinophilic asthma by regulating immune balance of Th1/2 and Treg/Th17 cells

**DOI:** 10.3389/fphar.2022.978421

**Published:** 2022-10-14

**Authors:** Yumei Zhou, Linhan Hu, Honglei Zhang, Haiyun Zhang, Juntong Liu, Xiaoshan Zhao, Ji Wang, Qi Wang

**Affiliations:** ^1^ School of Chinese Medicine, National Institute of TCM Body Constitution and Preventive Medicine, Beijing University of Chinese Medicine, Beijing, China; ^2^ School of Traditional Chinese Medicine, Southern Medical University, Guangzhou, Guangdong, China

**Keywords:** GMK, Th1/Th2, Treg/Th17, cellular immune balance, eosinophilic asthma

## Abstract

The number of patients with allergic asthma is rising yearly, and hormonal drugs, such as dexamethasone, have unique advantages and certain limitations. In the treatment of allergic diseases especially allergic asthma, increasing the percentage or the function of immunosuppressive cells, such as Treg cells, may achieve a good effect. On the basis of good clinical results, we found that Guominkang (GMK) especially high-concentration GMK can achieve a similar effect with dexamethasone in controlling the symptoms of allergic asthma and inhibiting inflammation of allergic asthma. In our study, GMK can inhibit the recruitment of inflammatory cells, decrease mucus production, and reduce airway resistance. Besides, GMK can reconstruct the cellular immune balance of Th1/2 and Treg/Th17 cells. Metabolome results show that DL-glutamine, L-pyroglutamic acid, prostaglandin b1, prostaglandin e2, and 3,4-dihydroxyhydrocinnamic acid are the metabolic biomarkers and are associated with Th1/2 and Treg/Th17 cell balance. GMK can also change the gut microbiota in the allergic asthma mouse model. The genus*_Muriculum*, genus*_(Clostridium) GCA900066575*, genus*_klebsiella*, *genus_Desulfovibrio*, genus*_*Rikenellaceae *RC9 gut group*, family*_*Chitinophagaceae*,* family*_*Nocardioidaceae, and genus*_Corynebacterium* are gut microbiota biomarkers treated by GMK. Among these biomarkers, genus*_Muriculum* is the gut microbiota biomarker associated with Th1/2 and Treg/Th17 cell balance. Interestingly, we first found that DL-glutamine, L-pyroglutamic acid, prostaglandin b1, prostaglandin e2, and 3,4-dihydroxyhydrocinnamic acid are all associated with genus*_Muriculum.* GMK will be a new strategy for the treatment of eosinophilic asthma, and biomarkers will also be a new research direction.

## Introduction

Asthma is a chronic inflammatory disease with high incidence rate, which can cause more than 250000 deaths every year (Fahy, 2015). Epidemiological studies showed that environmental risk factors (such as respiratory viral infections (Busse et al., 2010) and air pollutants (Peden, 2005)) and increasing urbanized lifestyles (including reduced exposure to microbes or their products (Braun-Fahrländer et al., 2002)) are factors inducing allergic asthma. Among the various forms of asthma (e.g., caused by allergens, air pollution, exercise, aspirin, and cold), allergic asthma is the most prevalent and can be induced by allergens, such as peanut, house dust mite (HDM), pollen, and animal dander. Inhaled corticosteroids (ICS) and long acting *β* Agonists are the basic treatment of asthma, which can effectively control the symptoms of allergic asthma. However, these therapies are not suitable for all patients with allergic asthma because some patients develop severe asthma. Severe asthma is characterized as difficulty in drug control, recurrent attacks, and chronic airflow obstruction (Lloyd and Hawrylowicz, 2009). Acute exacerbation of allergic asthma has a huge impact on both adults and children. At the same time, it brings a huge economic burden to patients with allergic asthma and endangers public health. According to the regularly revised global Asthma Initiative, few drugs are suitable for new biological agents or treatment schemes such as allergen specific immunotherapy (Duan et al., 2004; Sakaguchi et al., 2008).

Allergic asthma is predominantly divided into two inflammatory subtypes caused by T helper (Th) cells, i.e., Th2-high and Th2-low. The Th2-high subtype are associated with Th2 subtype cytokines such as IL (interleukin)-4, IL-5, and IL-13, and are characterized by airway eosinophilic infiltration. In allergic asthma, airway eosinophilia and goblet cell metaplasia are predominantly induced by IL-5 and IL-13, respectively (Finkelman et al., 2010; Chung, 2015). IL-4 has a certain correlation with sensitization and IgE production. IL-5 participates in eosinophil survival, and IL-13 affects the development and reorganization of airway hyperresponsiveness (AHR) (Sun et al., 2020). Studies have shown that the pathogenesis of airway inflammation in allergic asthma is related to the imbalance of Th1/Th2 cells, and the main reason for the excessive differentiation of Th2 cells is the insufficient differentiation of Th1 cells. An important strategy for the treatment of allergic asthma is to induce allergen immune tolerance (Heffler et al., 2019).

Current treatments based on glucocorticoid inhalation can only control Th2-driven eosinophil inflammation but cannot induce immune tolerance (Dhami et al., 2017). Foxp3+ Treg cells are critical for maintaining immune homeostasis in allergic asthma, and can inhibit inflammatory response (Joetham et al., 2007). Treg cells are a type of CD4^+^ T cell subpopulation, and the transcription factor Forkhead Box 3 (FOXP3), as a specific Treg cell maker, is essential to their function (Bullens et al., 2006; Finkelman et al., 2010). FOXP3 is the key to the differentiation of Treg cells. If FOXP3 gene is mutated, abnormal Treg cells will be produced. They lack regulatory function (Berker et al., 2017; Asayama et al., 2020). For patients with allergic asthma, the imbalance of Treg and Th cells in the process of allergic reaction has a certain impact on the development of asthma (Lee et al., 2009). IL-10 and TGF-β play an irreplaceable role in the regulation of allergic asthma. TGF-β can induce Foxp3 expression and Treg cell differentiation. IL-10 is also critical for the effective suppression of allergic reactions in the lung (Kudo et al., 2012). Recent studies showed that the IL-17A produced by Th17 cells promotes allergen-induced AHR through direct effects on airway smooth muscle (Gavin et al., 2007). The elevated levels of IL-17A are found in serum, sputum, and bronchoalveolar lavage (BAL) of patients with allergic asthma, and the concentrations of IL-17A are positively correlated with asthma severity at these sites (Baatjes et al., 2015; Zhao and Wang, 2018). Recent studies have shown that both Treg and Th17 cells have the ability to redifferentiate and belong to an unstable population (Tortola et al., 2019). Some treatment schemes are aimed at improving the symptoms of allergy and asthma. They all start from stimulating the proliferation of Treg cells to increase the number of Treg cells or restore the function of Treg cells (Hori et al., 2003). Therefore, increasing studies focused on stimulating Treg cell proliferation or inhibiting Th17 cell redifferentiation and changing the differentiation level of Treg or Th17 cells to rebuild the balance of Th17/Treg cells, which is correlated with asthma severity (Fontenot et al., 2003; Lin et al., 2007).

In the past few years, the combination of hormone therapy, such as ICS, with other medications, including a long-acting β2-agonist or a leukotriene modifier, is the first choice to treat allergic asthma, but not all patients achieve asthma control (Durrant and Metzger, 2010). A new drug that can induce immune tolerance and is expected to inhibit the recurrence of asthma is urgently needed. GMK is a drug prescribed by academician Qi Wang after many years of clinical experience and has achieved good clinical effect in treating allergic disease. In this study, we try to clarify the mechanism of GMK in treating eosinophilic asthma.

## Materials and methods

### Mice

Female BALB/c mice (age: 6–8 weeks old) were obtained from Beijing Vital River Laboratory Animal Technology Co. Ltd., Beijing, China. These mice were housed in pathogen-free conditions. All animal procedures were approved by Beijing University of Chinese Medicine Animal Care and Use Committee and conducted in accordance with AAALAC and IACUC guidelines.

### Preparation of GMK

**FIGURE 1:**
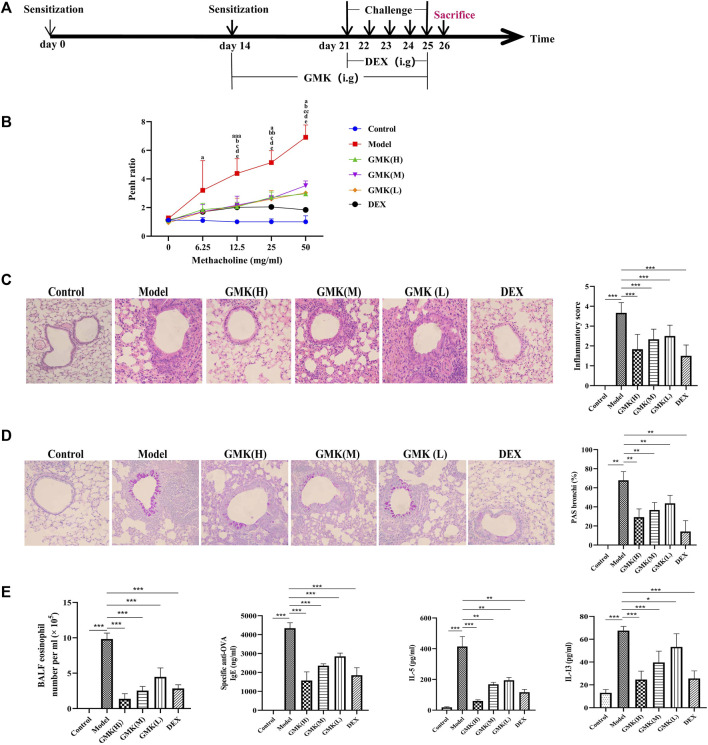
GMK can alleviate inflammation of eosinophilic asthma in BALB/c mice model. **(A)** Flow chart of eosinophilic asthma model construction in BALB/c mice. **(B)** Allergens airway hyperresponsiveness assessment by non-invasive methods. Mice (*n* = 4–5/group) were sensitized and challenged with OVA. a, *p* < 0.05 vs. Control; aa, *p* < 0.01 vs. Control; aaa, *p* ≤ 0.001 vs. Control. b, *p* < 0.05 vs. GMK(H); bb, *p* < 0.01 vs. GMK(H); bbb, *p* ≤ 0.001 vs. GMK(H). c, *p* < 0.05 vs. GMK(M); cc, *p* < 0.01 vs. GMK(M); ccc, *p* ≤ 0.001 vs. GMK(M). d, *p* < 0.05 vs. GMK (L); dd, *p* < 0.01 vs. GMK(L); ddd, *p* ≤ 0.001 vs. GMK(L); e, *p* < 0.05 vs. DEX; ee, *p* < 0.01 vs. DEX; eee, *p* ≤ 0.001 vs. DEX. **(C)** HE staining and inflammation score of lung tissue. **(D)** Typical PAS staining and the related corresponding score of lung tissue. **(E)** Specific OVA-IgE antibody detection in serum, eosinophil count in bronchoalveolar lavage fluid (BALF), IL-5 and IL-13 cytokines detection in BALF (*n* = 5–6/group). The experiments repeated 2–3 times in this study. *p < 0.05, **p < 0.01, ***p ≤ 0.001.

The four components of GMK used in our experiment are: Wu-Mei (Mume Fructus) 20 g, Chan-Tui (Cicadae Periostracum) 10 g, Fang-feng (Saposhnikoviae radix) 10 g, Ling-zhi (Ganoderma) 10 g, Shou-Wuteng (Caulis Polygoni Multiflori) 15 g, Tian-ma (Gastrodia elata Blume) 10g, they were named by Pharmacopoeia of China (2020). After soaked in deionized water for 30 min. The final concentration of the drug were GMK(H) group:19.5 g/kg/d, GMK(M) group: 9.75 g/kg/d, GMK(L) group:4.875 g/kg/d. More details can be found in references (Wu et al., 2019). DEX (Dexamethasone) was given 1 mg/kg/d, and it was dissolved in saline solution. The drug of GMK and DEX were given according to Figure 1A, GMK were given from day 14 to day 25, DEX was given from day 21 to day 25, they were all given once a day.

### Construction of eosinophilic asthma mouse model, administration of GMK, and detection of airway resistance

The eosinophilic asthma mouse model was induced by ovalbumin (OVA). Mice were sensitized on days 0 and 14 by intraperitoneal injection with 2 mg OVA (Sigma-Aldrich, Cat#A5503) and 2 mg Alum Adjuvant (Invitrogen, Cat#77161) dissolved in PBS. Mice inhaled 1%ova for 30 min from day 21 to day 25. BAL fluid (BALF) was collected 24 h after the last challenge.

On day 26, after challenge for 5 days, 0, 6.25, 12.5, 25, and 50 mg/ml methacholine (Sigma, Cat#A2251) was used to detect the enhanced pause (Penh) value. The airway resistance experiment was carried through the noninvasive measurement of airway hyper-responsiveness by whole-body plethysmography (WBP-4MR, TOW, China) as described previously (Li et al., 2009). The Penh ratio was used to represent the Penh measured (using methacholine divided by the mean Penh over a 5-min interval using PBS).

### Hematoxylin and eosin and periodic acid–schiff staining of lung tissue

Lung tissues were fixed in 4% paraformaldehyde, embedded into paraffin, and cut into 4 μm prepared sections. Lung tissues were stained by HE staining for cell infiltration detection or PAS for mucus production. As mentioned earlier, inflammatory cells and goblet cells were scored in at least three different areas of each lung section (Buzney et al., 2016).

### Percentage of Th1, Th2, Treg and Th17 cells and cytokines in BALF and specific OVA–IgE detection in Serum

Collecting spleen tissue and prepareing it into single cell suspension. Pay attention to the aseptic operation. For flow cytometry, cells were stimulated in complete RPMI containing 2 μl cocktail A (BD, Cat#550583) for 4 Construction of eosinophilic asthma mouse h, after blocked by Fc-receptor blocker (BD, Cat#513141) in 37°C for 40Construction of eosinophilic asthma mouse min, washed, and resuspended in 1 × PBS, and stained with FVS780 (BD, Cat#565388) to discriminate viable cells. The eBioscience Fix/Perm (Cat#00-5523-00) and BD Fix/Perm (Cat#554714) buffer kits were used to fix and permeabilize the cells. The intracellular staining Foxp3 (eBioscience, Cat#17-5773-82), IFN-γ (BD, Cat#557735), IL-4 (BD, Cat# 562915), or IL-17A (BD, Cat#564169) were used. Finally, we analyzed the data by the LSR Fortessa cell analyzer and Diva software (BD).

Multi-cytokine detection containing IL-4, IFN-γ, IL-5, IL-13, TGF-β, IL-6, IL-10, and IL17A was performed in accordance with the premixed AimPlex™ multiplex-assay kits (Cat#T2C0710709 and Cat#B111206). OVA-specific IgE in the serum was detected in accordance with the protocol (Cayman, Cat#500840).

### Detection of IFN-*γ*, IL-4, Foxp3, and RORγt mRNA levels by real-time PCR

\The transcription factors of IFN-γ, IL-4, FOXP3, and RORγt in lung tissue were detected, and the total RNA from the tissues was extracted by TRIzol (Invitrogen) in accordance with the manufacturer’s instructions. cDNA was synthesized by reverse transcription by using the first-strand cDNA synthesis kit (Servicebio, G3330) in accordance with the manufacturer’s instructions and used for real-time PCR assay performed in 1× SYBR green qPCR master mix (Servicebio, G3320) together with 0.2 mM forward and reverse primers. The amount of mRNA of the indicated genes after normalization of *β*-actin mRNA. The primers of GAPDH, Foxp3, RORγt, IFN-γ and IL-4 genes were as described as in a previous study (Zhou et al., 2022).

### Untargeted plasma metabolomics detection and analysis

In this study, UHPLC (1290 Infinity LC, Agilent Technologies) and quadrupole time-of-flight (AB Sciex TripleTOF 6,600) in Shanghai Applied Protein Technology Co., Ltd. were used to perform LC-MS/MS analysis. The orthogonal partial least-squares discriminant analysis was used to perform multivariate data analysis, and VIP >1 and *p* value <0.05 were used to screen metabolites’ significant changes. The Pearson correlation analysis with R package was used to determine the correlation between variables.

### Gut microbiota detection and analysis

The 16S rDNA amplicon sequencing was used to detect gut microbiota after extracting the total genome DNA by the CTAB/SDS method (Bansal et al., 2018). Sequencing libraries were generated using the NEB Next®Ultra™DNA Library Prep Kit for Illumina (NEB, United States) following the manufacturer’s recommendations, and index codes were added. The library was sequenced on the Illumina Miseq/HiSeq2500 platform. STAMP software was used to confirm the difference in abundance value, and LEfSe was used to conduct quantitative analysis of biomarkers in different groups.

### Correlation analysis of differential plasma metabolites and differential gut microbiota with Th1, Th2, Treg and Th17 cells in eosinophilic asthma

The Pearson correlation analysis with R package was performed to determine the correlation of variables, such as differential plasma metabolites; differential gut microbiota; Th1, Th2, Treg and Th17 cell percentages; specific OVA–IgE antibody; Penh value (Methacholine: 12.5, 25, and 50 mg/ml); and eosinophilic number in BALF.

### Statistical analysis

Use SPSS 20.0 software for statistical analysis of data. Data are presented as means ± SEM. Analysis of variance (ANOVA) with the Bonferroni correction for post hoc comparisons was used to test group differences, and the rank sum test was used for percentage analysis. *p* < 0.05 was considered statistically significant. The Pearson correlation analysis was used to analyze the correlation of differential metabolites and differential gut microbiota with Th1/2 and Treg/Th17 cells.

## Results

### GMK plays a certain role in relieving allergic inflammation in eosinophilic asthmatic mouse model

The allergic asthma model in BALB/c mice with eosinophil infiltration was constructed as shown in Figure 1A. The Penh value, which reflected airway resistance, indicated that GMK could significantly decrease the Penh value. The high-concentration GMK, GMK(H) had good effect, whereas the positive drug group, i.e., Dexamethasone (DEX) group, had the best effect (Figure 1B). The same effect could be seen in Figures 1C,D. GMK especially the (GMK(H)) group could achieve improved effect, whereas DEX had the best effect in inhibiting allergic inflammation, such as inflammatory cell infiltration (Figure 1C) and mucus production (Figure 1D). Interestingly, in the detection of specific OVA–IgE, IL-5, and IL-13 antibodies and eosinophilic number (Figure 1E), DEX had the best effect, and compared with the middle- and low-concentration GMK, GMK(H) had the best effect.

### GMK can reconstruct Th1/2 cellular balance in eosinophilic asthma

**FIGURE 2:**
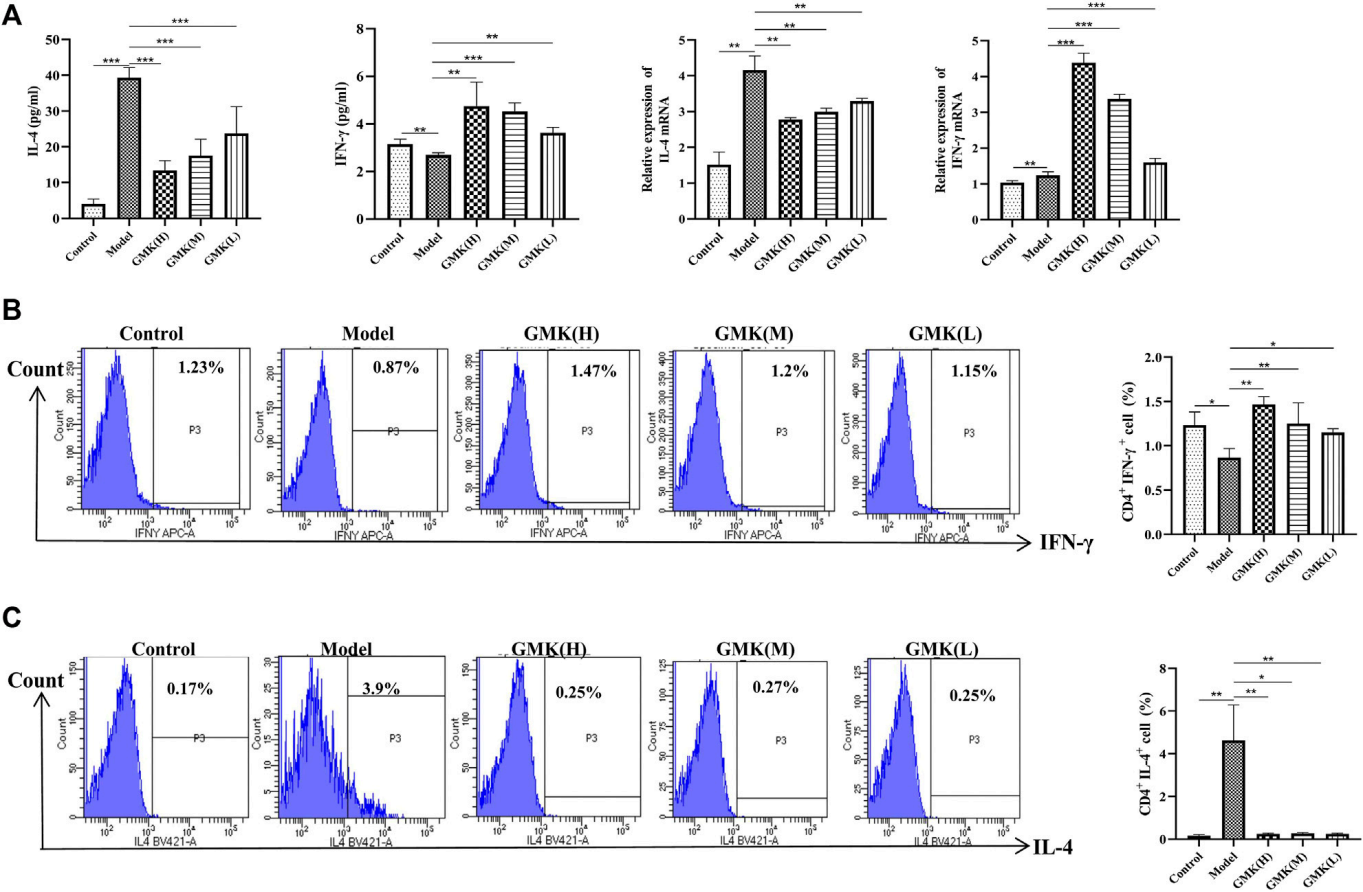
Th1, Th2 cells and their related cytokines and gene detection. **(A)** Th1 and Th2 cells related cytokines detection in BALF and the IL-4 and IFN-γ mRNA expression in lung tissue analyzed by real-time RT-PCR. **(B,C)** Flow cytometry detection of Th1 and Th2 cells in spleen tissue, results was all represent as mean±SEM (*n* = 5–6/group). CD3^+^CD4^+^ cells were gated, then IFN-γ+ cells were gated for Th1 cells. CD3^+^CD4^+^ cells were gated, then IL-4^+^ cells were gated for Th2 cells. The experiments repeated 3 times in this study. *p < 0.05, **p < 0.01, ***p ≤ 0.001.

The Th1/2 cell balance was destroyed in the eosinophilic asthma model but reconstructed after GMK treatment especially in the GMK(H) group (Figures 2B,C **)**. The cytokines of IL-4 and IFN-γ in serum and the mRNA expression levels of IL-4 and IFN-γ in lung tissue had the same effect **(** Figure 2A **)**. Surprisingly, DEX did not have the same effect and could decrease Th2 cell percentage and related cytokines, such as IL-4 and IFN-γ mRNA expression, but can not increase Th1 cell percentage and the related cytokine and mRNA expression, such as IFN-γ.

### Treg/Th17 cellular immune balance is reconstructed after GMK treatment

**FIGURE 3:**
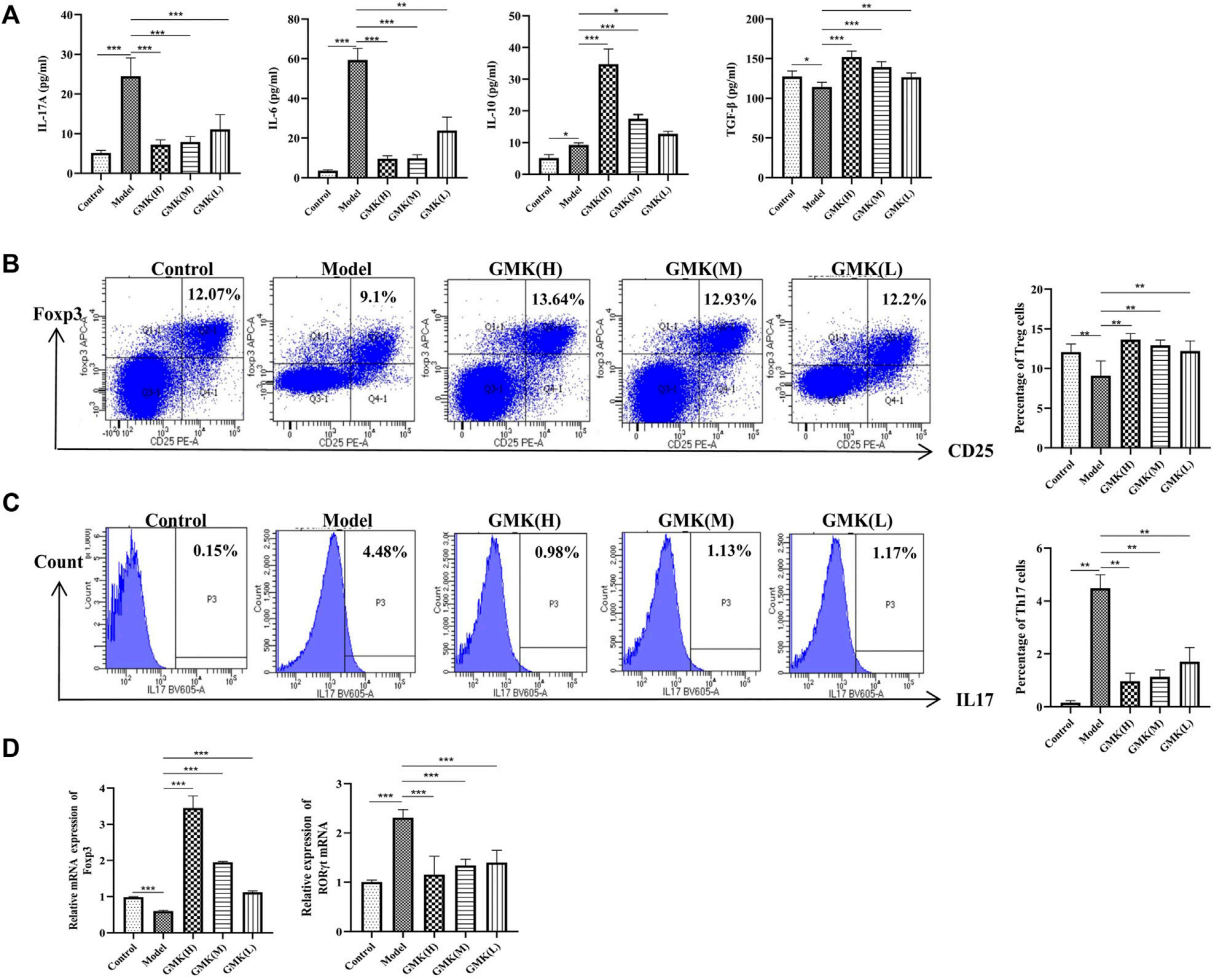
Treg, Th17 cells and their related cytokines and mRNA detection. **(A)** Treg and Th17 cells related cytokines detection in BALF. **(B,C)** Percentage detection of Treg and Th17 cells in spleen tissue. **(D)** The Foxp3 and RORγt mRNA relative expression in the lung tissue analyzed by real-time RT-PCR, results was represent as mean±SEM (*n* = 5–6/group). CD3^+^CD4^+^ cells were gated, then CD25^+^Foxp3^+^ cells were gated for Treg cells. CD3^+^CD4^+^ cells were gated, then IL-17^+^ cells were gated for Th17 cells. The experiments repeated 3 times in this study.*p < 0.05, **p < 0.01, ***p ≤ 0.001.

The allergic asthma model group showed a significant decrease in the percentage of Treg cells. In contrast, the percentage of Treg cells increased significantly in the GMK groups, especially in the GMK (H) group **(** Figures 3B,C **)**. Interestingly, GMK groups especially the GMK(H) group had decreased Th17 cell detection. The mRNA expression of Foxp3 and RORγt in lung tissue is consistent with the detection results of Treg and Th17 cells in Figure 3D. The cytokine detection result showed that GMK treatment decreased IL-17A and IL-6 levels and increased IL-10 and TGF-β levels **(** Figure 3A **)**. DEX also achieved the same effect as GMK(H) in the detection.

### Plasma metabolites were changed after GMK treatment

**FIGURE 4:**
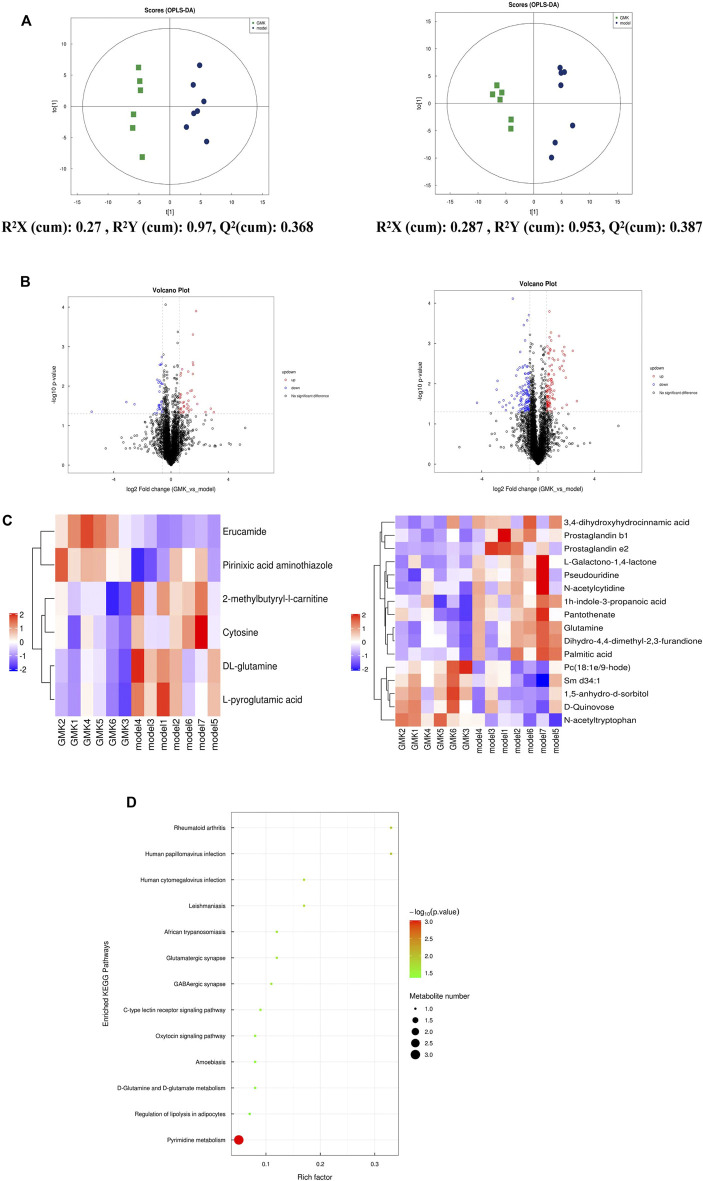
(Continued).

We studied the plasma metabolism after GMK treatment to explore the mechanism of GMK on immune balance reconstruction in eosinophilic asthma. As shown in Figure 4A, metabolites were detected in the positive (POS) and negative (NEG) modes (i.e., 0.3 < Q^2^ = 0.368 < 0.5 in POS mode and 0.3 < Q^2^ = 0.387 < 0.5 in NEG mode), which indicated that the model was stable. A total of 5,058 and 4,387 metabolites in the POS and NEG modes, respectively, were detected. Six of these metabolites were significantly expressed in POS mode and 16 were significantly expressed in NEG mode (Table 1). Differential metabolites with FC > 1.5 or FC < 0.67 and *p* value <0.05 were visualized using a volcano graph in Figure 4B. The results of the hierarchical clustering analysis of differential metabolites, 6 and 16 metabolites in POS and NEG modes respectively are shown in Figure 4C. In the KEGG pathway analysis, most differential metabolites were enriched to pyrimidine metabolism and d-glutamine and d-glutamate metabolism pathway (Figure 4D).

**TABLE 1:** Differentiated plasma metabolites between model and GMK groups.

Name	ESI	VIP	Fold Change	*p* value
Erucamide	+	10.05189	3.374263664	0.000125
L-pyroglutamic acid	+	3.846949	0.830618952	0.004358
DL-glutamine	+	2.412555	0.832989231	0.005224
2-methylbutyryl-l-carnitine	+	1.272464	0.799934156	0.011032
Pirinixic acid aminothiazole	+	1.030683	1.77173329	0.02643
Cytosine	+	1.239749	0.849232667	0.03137
N-acetyltryptophan	−	1.779763	1.402748814	0.001626
Glutamine	−	3.114074	0.760124438	0.004224
1,5-anhydro-d-sorbitol	−	2.085506	1.391837849	0.006918
Dihydro-4,4-dimethyl-2,3-furandione	−	1.167511	0.78160733	0.007372
L-Galactono-1,4-lactone	−	1.725859	0.684250598	0.013781
Pc(18:1e/9-hode)	−	2.2884	1.885403306	0.013944
Prostaglandin e2	−	3.424605	0.130341353	0.014098
Prostaglandin b1	−	1.169405	0.514396644	0.014977
Pantothenate	−	1.852229	0.745324355	0.017882
1h-indole-3-propanoic acid	−	1.479907	0.595061082	0.01828
Pseudouridine	−	1.240415	0.78508799	0.030125
Sm d34:1	−	2.112093	1.383823251	0.031022
3,4-dihydroxyhydrocinnamic acid	−	1.449743	0.689174528	0.036569
d-Quinovose	−	1.304085	1.279815081	0.039916
N-acetylcytidine	−	1.363898	0.736552224	0.047463
Palmitic acid	−	15.57049	0.78886185	0.049912

### Biomarkers associated with Th1/2 cell or Treg/Th17 cellular immune balance at the metabolic and gut microbiota levels

**FIGURE 5:**
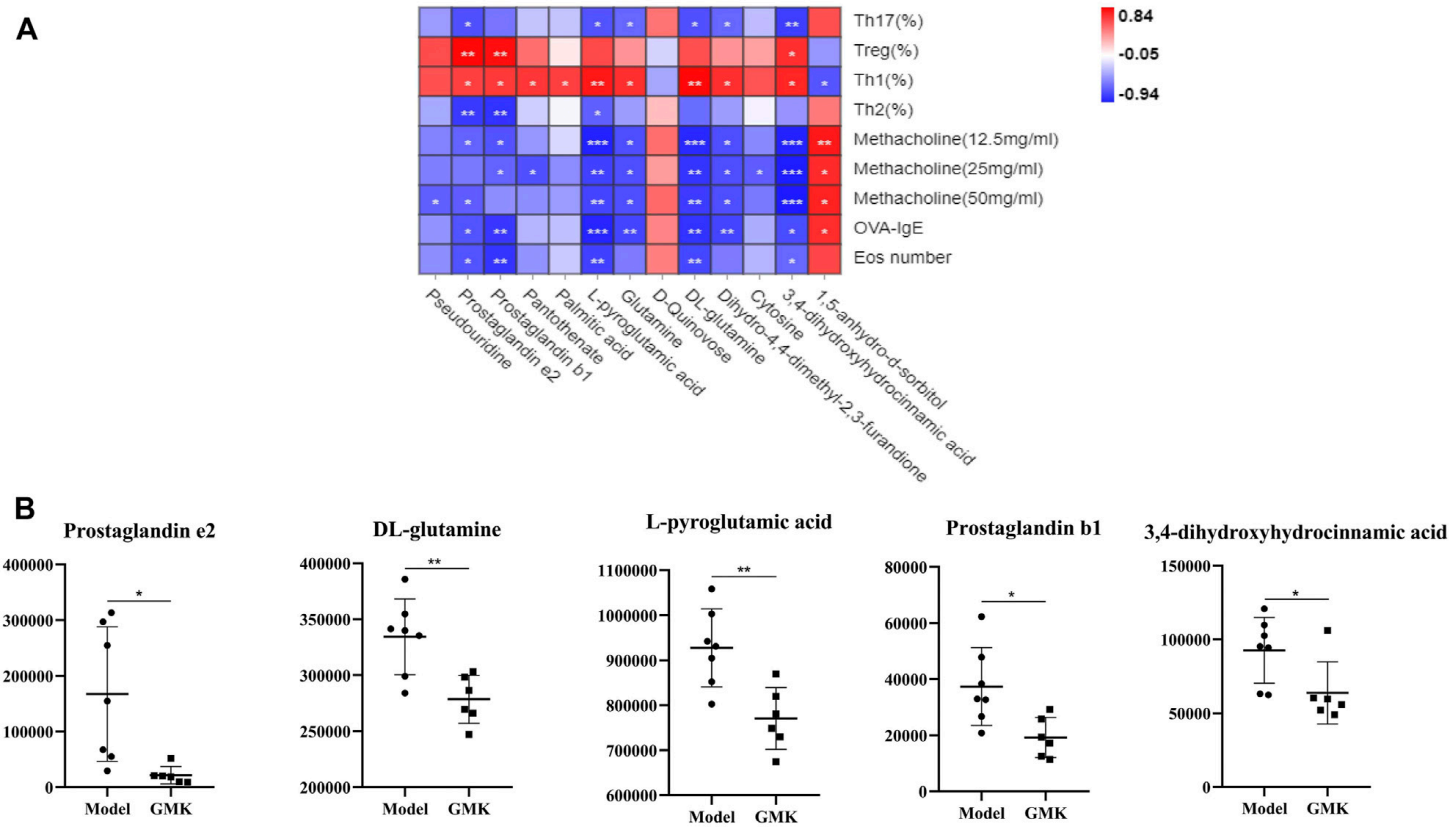
Correlation analysis between differential metabolites and Th1/2, Treg/Th17 cells balance treated by GMK in eosinophilic asthma. **(A)** Pearson correlation analysis between differential metabolites and Th1/2, Treg/Th17 immune balance, Penh value, OVA-IgE antibody and eosinophil number in BALF compared between GMK(H) and model group. **(B)** Metabolic biomarkers closely related to Th1/2 and Treg/Th17 cell balance in eosinophilic asthma (*n* = 7/group). *p < 0.05, **p < 0.01, ***p ≤ 0.001.

The Pearson correlation analysis was used to analyze the correlation and further explore the immune mechanism of GMK in treating eosinophilic asthma at the metabolic level (Figures 5A,B). The plasma metabolites in the GMK(H) and eosinophilic asthma mice model groups were detected. Among the differential metabolites, DL-glutamine, L-pyroglutamic acid, prostaglandin b1, prostaglandin e2 and 3,4-dihydroxyhydrocinnamic acid were the metabolic biomarkers, they were all connected to OVA-IgE, Penh value (Mch:12.5,25,50 mg/ml), Eos number, Th1/2 and Treg/Th17 cell balance.

**FIGURE 6:**
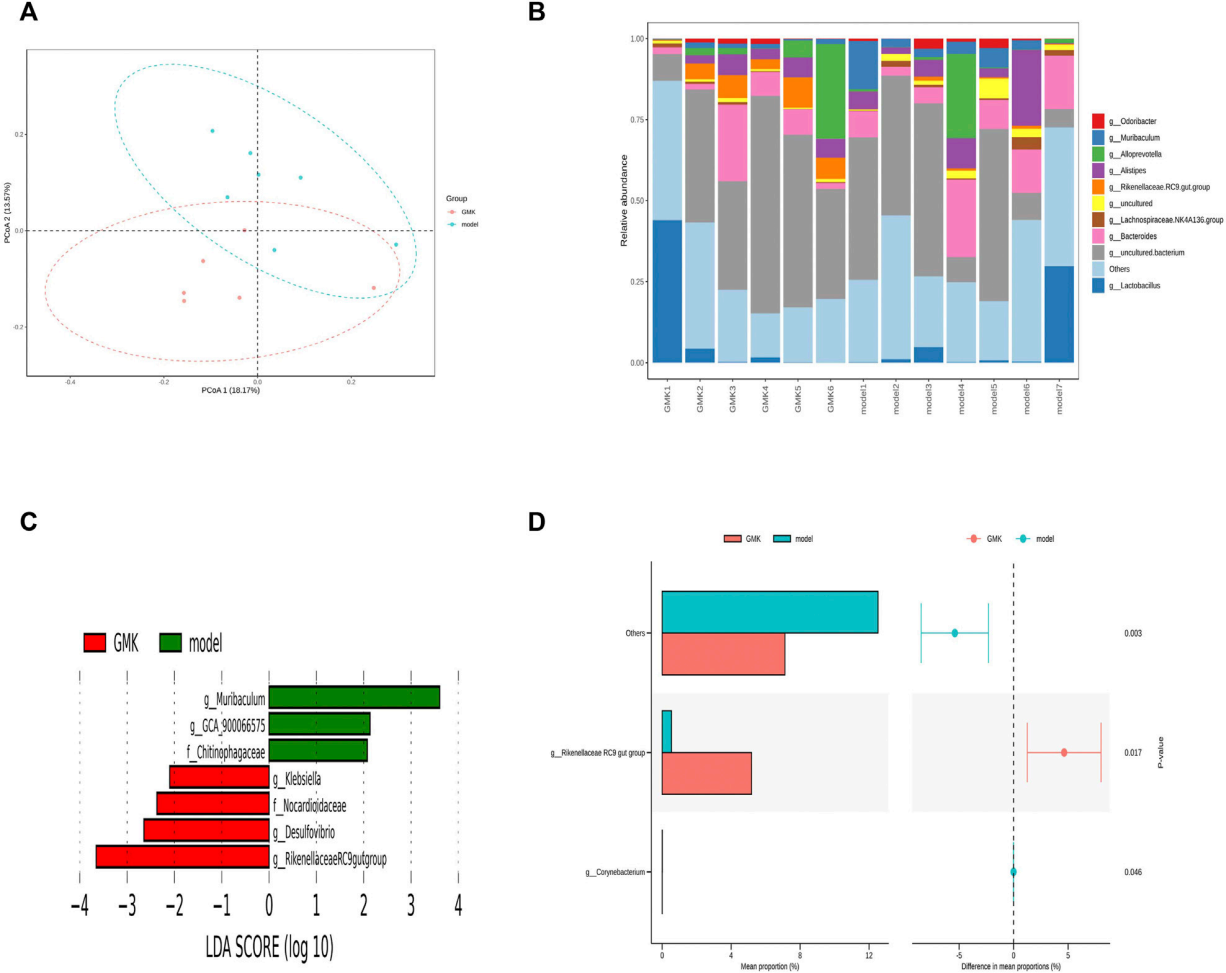
Gut microbiota Biomarker detection after GMK treated. **(A)** Principal coordinate analysis (PCoA) of gut microbiota in GMK treated group and eosinophilic asthma model group; **(B)** The top 10 gut microbiota at genus level between GMK treated group and model group; **(C)** Lefse analysis of differential gut microbiota between GMK treated group and model group (LDA >2 was used); **(D)** STAMP analysis of differential gut microbiota between GMK(H) treated group and model group (*p* < 0.05 was used), *n* = 7/group.

GMK was treated by intragastric administration to further investigate whether GMK could change gut microbiota, and 16S rDNA amplicon sequencing analysis was performed in the study. The gut microbiota was separated significantly (Figure 6A) and the top 10 relative abundance at genus level (Figure 6B). The LDA Effect Size (LEfSe) and STAMP analyses were used to examine the differential gut microbiota between GMKand model groups. LDA value >2 was used in the LEfSe analysis, and *p* value <0.05 was used in STAMP analysis. Genus*_Muriculum*, genus*_(Clostridium) GCA900066575*, genus*_klebsiella*, genus*_Desulfovibrio*, genus*_*Rikenellaceae *RC9 gut group*, family*_*Chitinophagaceae*,* family*_*Nocardioidaceae, and genus*_ Corynebacterium* were gut microbiota biomarkers after GMK treatment as shown in Figures 6C,D.

**FIGURE 7:**
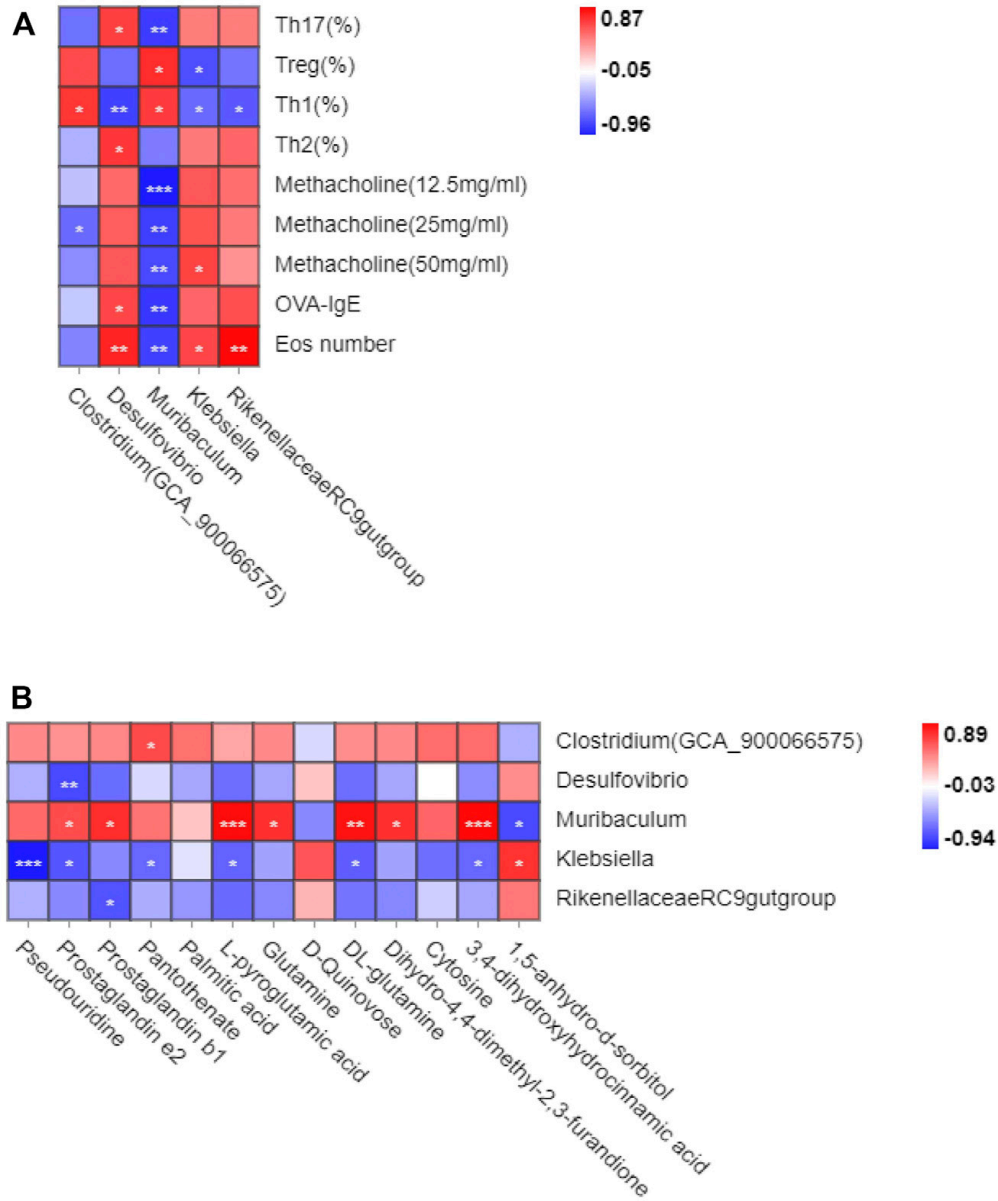
Correlation analysis between gut microbiota and Th1/2, Treg/Th17 cell balance in eosinophilic asthma. **(A)** Correlation analysis between differential microbiota and Th1,Th2, Treg and Th17 cell percentage, Penh value, OVA-IgE antibody and eosinophil number in BALF. **(B)** Correlation analysis of diffenential metabolites and gut microbiota biomarker treated by GMK (*n* = 5–6/group).

The Pearson correlation analysis was used in this study to investigate whether gut microbiota changes were related to the reconstruction of immune balance. The genus*_* (*Clostridium*)*GCA900066575*, genus*_Desulfovibrio*, genus*_Muriculum*, and genus*_* Rikenellaceae *RC9 gut group* were all associated with Th1 cells, and genus*_Desulfovibrio* was associated with Th2 cells. These cells were important in the Th1/2 cell balance. Genus*_Muriculum* and genus*_klebsiella* were associated with Treg cells, and genus*_Desulfovibrio* and genus*_Muriculum* related to Th17 cells. These cells were essential in the Treg/Th17 immune balance. Among them, genus*_Muriculum* is the biomarker based on the OVA-IgE, Penh value (Mch:12.5, 25, 50 mg/ml), Eos number, Th1, Th2, Treg and Th17 cell percentage, which indicated that genus*_Muriculum* is the gut microbiota biomarker interrelated to Th1/2 and Treg/Th17 cell balance (Figure 7A).

Gut microbiota and plasma metabolites play essential roles in GMK treatment. Genus*_*(*Clostridium*)*GCA900066575* was associated with pantothenate, genus*_ Desulfovibrio* was associated with prostaglandin e2, and genus*_Muriculum* was associated with prostaglandin e2, prostaglandin b1, L-pyroglutamic acid, glutamine, DL-glutamine, dihydro-4,4-dimethyl-2,3-furandione, 3,4-dihydroxyhydrocinnamic acid, and 1,5-anhydro-d-sorbitol. Genus*_klebsiella* was associated with pseudouridine, prostaglandin e2, pantothenate, L-pyroglutamic acid, DL-glutamine, 3,4- dihydroxyhydrocinnamic acid, and 1,5-anhydro-d-sorbitol. Genus*_*Rikenellaceae *RC9 gut group* was associated with prostaglandin b1 (Figure 7B). Very interesting, the metabolites biomarker of prostaglandin e2, prostaglandin b1, L-pyroglutamic acid, DL-glutamine, 3,4-dihydroxyhydrocinnamic acid are all associated with genus*_Muriculum.*

## Discussion

Many studies have proved that the inflammation induced by a variety of Th cells, including Th1, Th2, Th9, Th17 and Th22 cells and their specific cytokines, has a certain correlation with the onset and development of allergic asthma. Th2 cells should not be neglected in the pathogenesis of allergic inflammation due to an imbalance of Th1/Th2 response (Hartl et al., 2007). More and more studies have shown that the root causes of Th2 response enhancement and allergic asthma are insufficient differentiation and functional defects of Treg cells. One study (Hanania, 2008) reported that patients with allergic asthma have fewer Treg cells in the peripheral blood than nonasthma normal ones. Another study proved that young patients with asthma and ICS treatment have fewer Treg cells in the lungs than normal ones, but this treatment fails to suppress pulmonary Th2 responses (Sun et al., 2021). Treg cells have an immuno suppressive function and are predominantly adjusted to the Foxp3 gene (Kim et al., 2022). Th17 cells have a certain correlation with the pathogenesis of asthma. This cell regulates eosinophil and neutrophil inflammation. (Maggi et al., 2021). Therefore, Th1, Th2, Treg, and Th17 cells play critical roles in maintaining immune homeostasis. Reducing immune response caused by Th2 or Th17 cells, promoting differentiation of Treg cells, or increasing the numbers of Th1 and Treg cells to rebuild the immune balance of Th1/2 or Treg/Th17 cells are new strategies to treat allergic asthma.

GMK, which regulates body constitution, is a classic prescription in the field of allergy field for academician Qi Wang. Regulating body constitution in allergic asthma disease means regulating immune homeostasis in patients with allergic asthma. GMK plays a role in reducing Ag-specific IgE, Ag-induced T-cell proliferation, and mast cell histamine release in treating allergic rhinitis (Zhou et al., 2018). Eosinophilia is a hallmark of allergic airway inflammation, and eosinophils can participate in many immune processes, such as Ag presentation and release of stored proinflammatory mediators (e.g., cytokines, chemokines, reactive oxygen species, lipid mediators, and granule proteins) (Heul et al., 2019). In the present study, an allergic asthma mouse model predominantly infiltrated by eosinophils with OVA and aluminum hydroxide is successfully constructed. GMK has a critical role in inhibiting eosinophil infiltration by reducing the eosinophil number and inhibiting related cytokines, such as IL-5 and IL-13. Imbalances in Th1/2 and Treg/Th17 cells exist in the allergic asthma model. The role of GMK in regulating immune balance is first evaluated. GMK can inhibit eosinophilic asthma by decreasing eosinophil number, decreasing inflammation index, and reducing airway resistance. GMK can also make the immune balance of Th1/2 and Treg/Th17 cells normal. GMK has the same function of DEX in suppressing allergic asthma and has unique advantages in immune balance reconstruction especially in increasing the percentage of Th1 cells. This result may be related to the way the model is constructed and needs to be validated in combination with clinical practice.

In the plasma metabolism detection, DL-glutamine, L-pyroglutamic acid, prostaglandin b1, and 3,4-dihydroxyhydrocinnamic acid are initially identified as biomarkers associated with Th1/2 and Treg/Th17 immune balance on the basis of reducing eosinophil number, inhibiting Ag-specific IgE antibody, and reducing inflammation induction. Glutamine can suppress allergic airway inflammation through the upregulation of MAPK phosphatase-1, which is consistent with our results (Kim et al., 2022). ILC2s predominantly participate in the physiopathology of allergic diseases especially eosinophilic asthma (Maggi et al., 2021). Prostaglandin E2 can inhibit the production of IL-5 and IL-13 and the amplification of ILC2 *in vitro* (Zhou et al., 2018). At present, there is no report that prostaglandin b1 and 3,4-dihydroxyhydrocinnamic acid are involved in the treatment of allergic asthma. They may be a new strategy for the treatment of allergic asthma. While until now, there are few study focus on the relationship between gut microbiota and plasma metabolites, and the relationship between gut microbiota, plasma metabolites and the function of T cells, they will be a new research direction for us.

Intestinal microbiota can affect the immune response and physiology of allergic asthma, and also affect the activity and number of T cell subsets including Th1, Th2, Th17 and effector/memory T lymphocytes (Heul et al., 2019). Study shows *Klebsiella*/*Bifidobacterium* in early life is correlate with later development in paediatric allergy (Low et al., 2017). Genus_ *Corynebacterium* in the nasopharynx (NP) impacts severity of lower respiratory infection and risk of asthma development (Teo et al., 2015). T lymphocyte activation is dependent on glutamine, which is essential nutrient in the activation of naive T cells (Carr et al., 2010). Prostaglandin e2 (PGE2) can regulate immune response, such as it can modulate local attraction and degranulation of mast cells (Hu et al., 1995; Weller et al., 2007), it plays essential role in activation and migration of DC cells (Kalinski et al., 1998), it can also inhibit activation and expansion of naive T cells (Muthuswamy et al., 2010). Moreover, PGE2 can influence Th1 and Th2 response (Betz and Fox, 1991; Kapsenberg et al., 1999), differentiation of Treg and Th17 cells (Muthuswamy et al., 2008; Boniface et al., 2009), which is important in allergic asthma. All these results is consistent with our results and can support our preliminary conclusions, while it still need further experimental verification in the future study.

The oral administration of *Clostridium* can reduce OVA-induced allergic airway inflammation in a mouse model, whereas it is only associated with airway resistance and Th1 cells in our study (Juan et al., 2017). At present, we focus our research on the biomarkers of genus_*Muriculum*, genus_*klebsiella*, and genus_*Desulfovibrio*. Genus_*Desulfovibrio* may influence Treg/Th17 balance in ulcerative colitis (Cui et al., 2018). Interestingly, DL-glutamine, L-pyroglutamic acid, prostaglandin b1, prostaglandin e2, and 3,4-dihydroxyhydrocinnamic acid are associated with genus_*Muriculum*; prostaglandin e2 is associated with genus_*Desulfovibrio*; and DL-glutamine, L-pyroglutamic acid, prostaglandin b1, prostaglandin e2, and 3,4-dihydroxyhydrocinnamic acid are associated with genus_*klebsiella*. The above results are studied, and they may be a new strategy to treat allergic asthma. While, until now there are none research about the DL-glutamine, L-pyroglutamic acid, prostaglandin b1, prostaglandin e2, and 3,4-dihydroxyhydrocinnamic acid associated with genus_*Muriculum*. 1,5-anhydro-d-sorbitol is associated with Treg/Th17 cell balance in allergic asthma mouse model (Zhou et al., 2022). There are none research based on the penh value associated them. Our future research will be conducted on the above basis. Given the limitations, in the future study, it should be combined with clinical results. Besides that, the research will also focus on other allergic asthma model like neutrophilic asthma induce by HDM or other inducer.

## Data Availability

The datasets presented in this study can be found in online repositories. The names of the repository/repositories and accession number(s) can be found in the article/supplementary material.
