# Supplementary material for: Guominkang formula alleviate inflammation in eosinophilic asthma by regulating immune balance of Th1/2 and Treg/Th17 cells
Source: Front Pharmacol. 2022 Oct 14;13:978421. doi: 10.3389/fphar.2022.978421 (PMC9624229; doi:10.3389/fphar.2022.978421)
Supplement: Supplementary file 4 [file Table2.DOCX]

**Figure Legends**

**
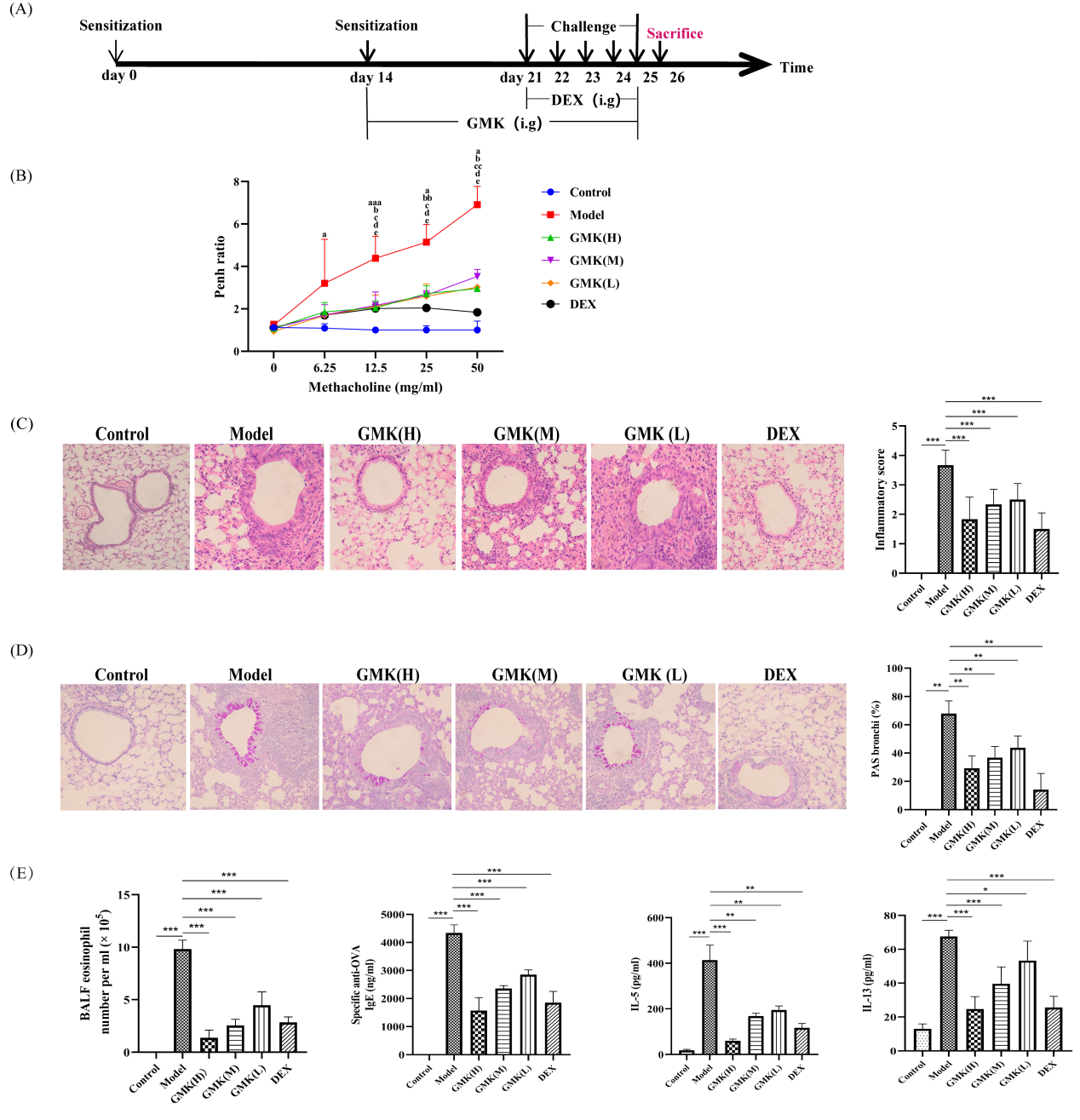
**

**Figure 1 Guominkang can alleviate inflammation of eosinophilic asthma in BALB/c mice model.** (A) Flow chart of eosinophilic asthma model constructing in BALB/c mice. (B) Allergens airway hyperresponsiveness assessment by non-invasive methods Mice (n=4-5/group) were sensitized and challenged with OVA. a, *p* < 0.05 vs Control; aa, *p* < 0.01 vs Control; aaa, *p* ≤ 0.001 vs Control. b, *p* < 0.05 vs GMK(H); bb, *p* < 0.01 vs GMK(H); bbb, *p* ≤ 0.001 vs GMK(H). c, *p* < 0.05 vs GMK(M); cc, *p* < 0.01 vs GMK(M); ccc, *p* ≤ 0.001 vs GMK(M). d, *p* < 0.05 vs GMK (L); dd, *p* < 0.01 vs GMK(L); ddd, *p* ≤ 0.001 vs GMK(L). (C) HE staining and inflammation score of lung tissue. (D) Typical PAS staining and the related corresponding score of lung tissue. (E) Specific OVA-IgE antibody detection in serum, eosinophil count in bronchoalveolar lavage fluid（BALF), IL-5 and IL-13 antibody detection in serum (n=5-6/group). * *p* < 0.05, ***p* < 0.01, *** *p* ≤ 0.001.

**
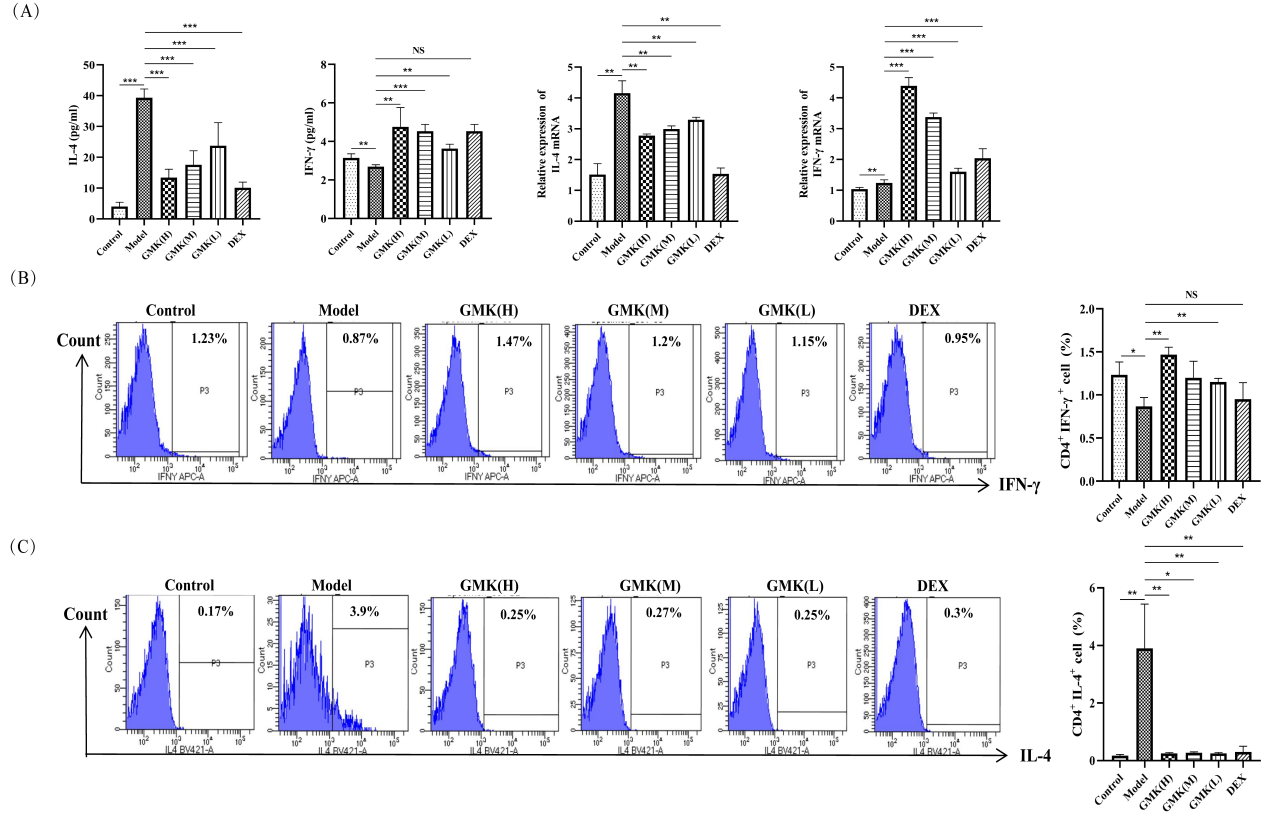
**

**Figure 2 Th1, Th2 cells and their related cytokines and gene detection.** (A) Th1 and Th2 cells related cytokines detection in serum. (B,C) Flow cytometry detection of Th1 and Th2 cells in spleen tissue. (D) The IL-4 and IFN-γ gene expression in lung tissue analyzed by real-time RT-PCR, results was all represent as mean +/- SEM (n=5-6/group). * *p* < 0.05, ***p* < 0.01, *** *p* ≤ 0.001.

**
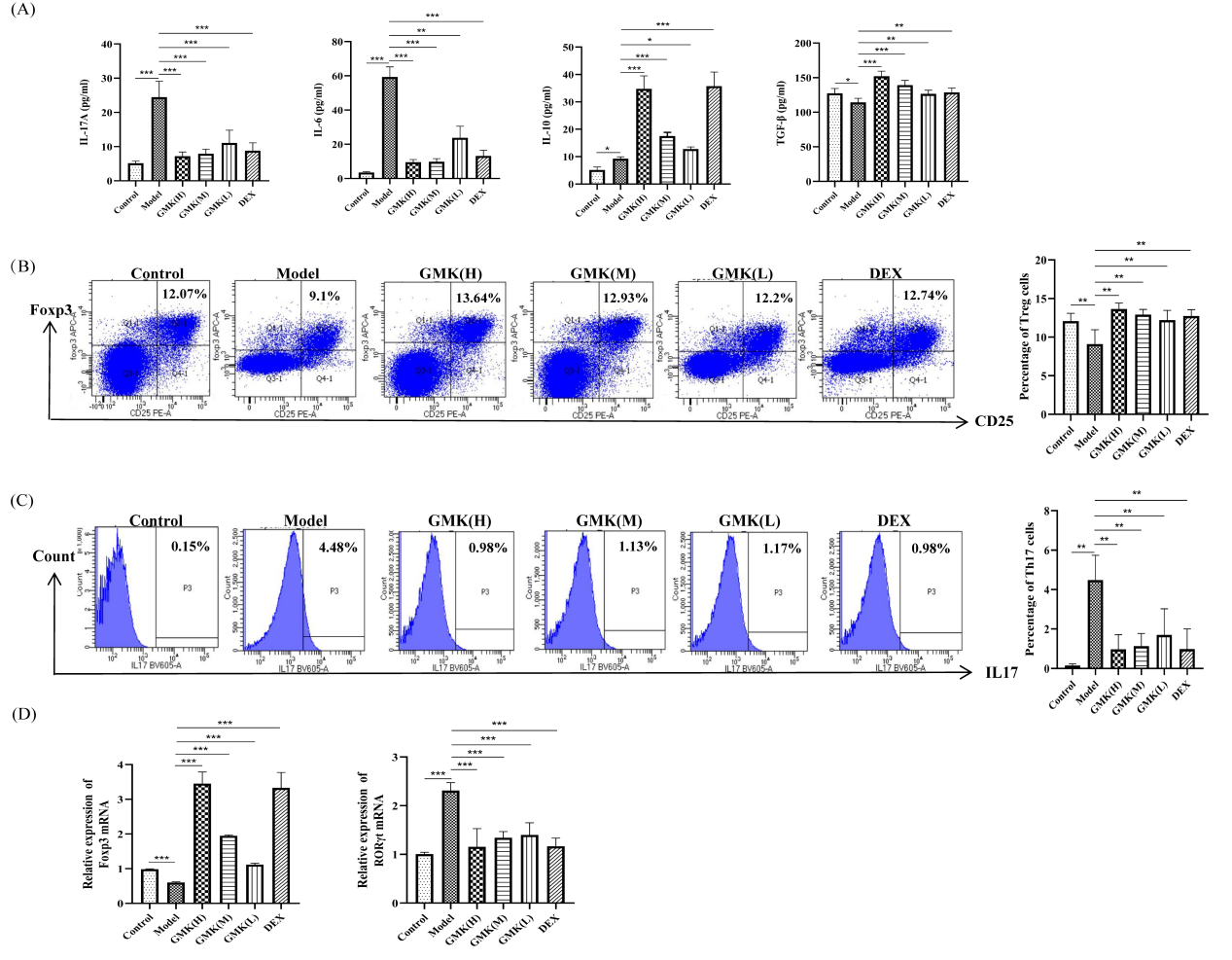
**

**Figure 3 Treg, Th17 cells and their related cytokines and gene detection.** (A) Treg and Th17 cells related cytokines detection in serum. (B,C) Flow cytometry detection of Treg and Th17 in spleen tissue. (D)The Foxp3 and RORγt gene expression in lung tissue analyzed by real-time RT-PCR, results was represent as mean +/- SEM (n=5-6/group). * *p* < 0.05, ***p* < 0.01, *** *p* ≤ 0.001.

**
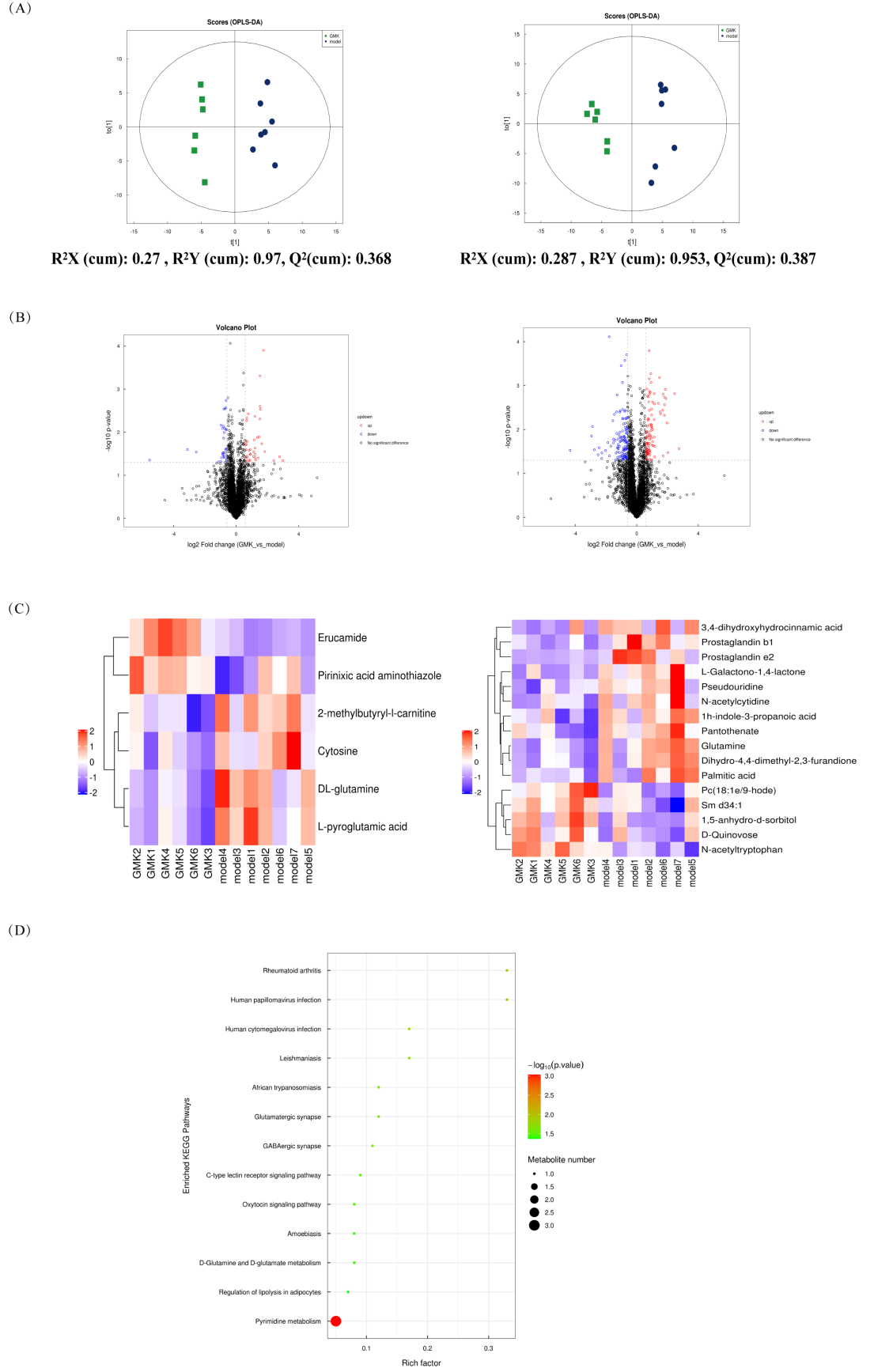
**

**Figure 4 Untargeted metabolomics of plasma detection treated by Guominkang.** (A) OPLS-DA score plots derived from UPLC-Q-TOF/MS in positive and negative ionization modes. (B) Volcano map in positive mode and negative mode. (C) The hierarchical cluster analysis of different metabolites between GMK and model group under positive and negative modes. (D) Enriched KEGG pathways based on significant different metabolites between GMK and model group (n=7/group).

**
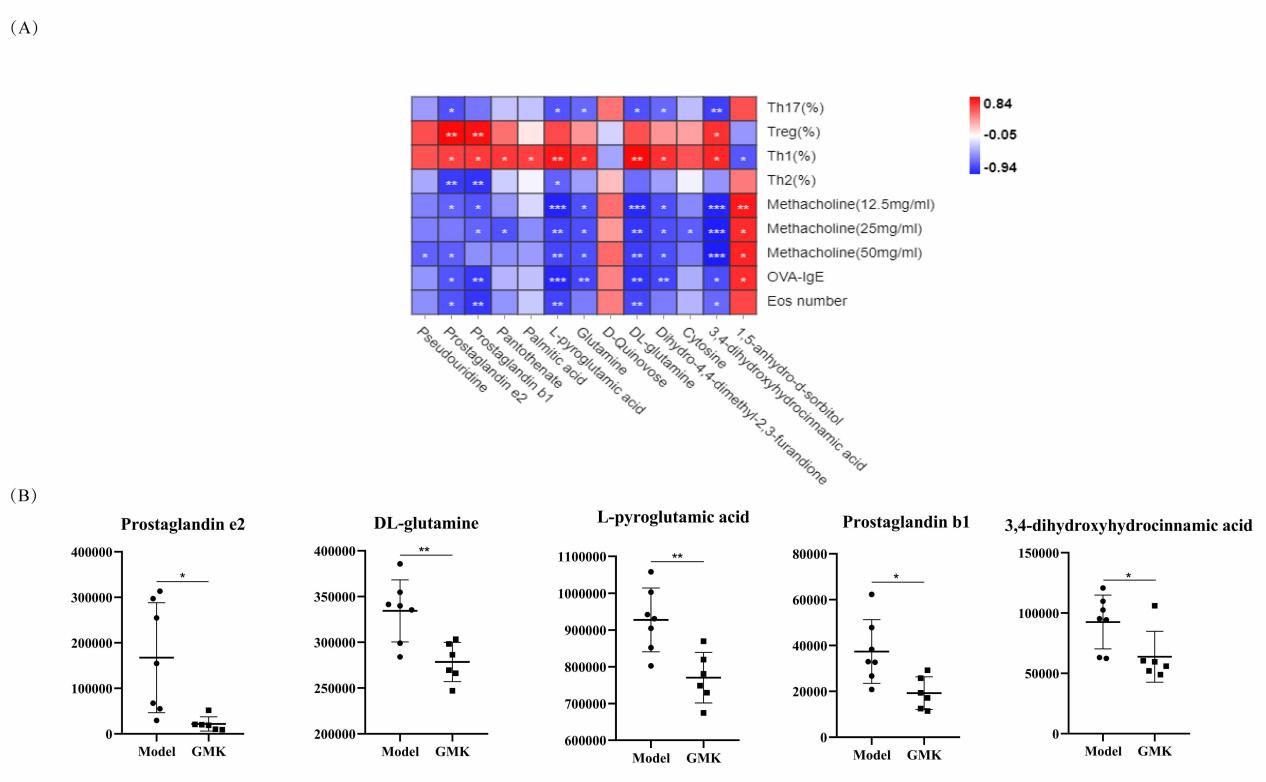
**

**Figure 5 Correlation analysis between differential metabolites and Th1/2, Treg / Th17 cells balance in eosinophilic asthma.** (A) Pearson correlation analysis between differential metabolites and Th1/2, Treg/Th17 immune balance, Penh value, OVA-IgE antibody and eosinophil number in BALF. (B) Metabolic biomarkers closely related to Th1/2 and Treg/Th17 cell balance in eosinophilic asthma (n=7/group). * *p* < 0.05, ***p* < 0.01, *** *p* ≤ 0.001.

**
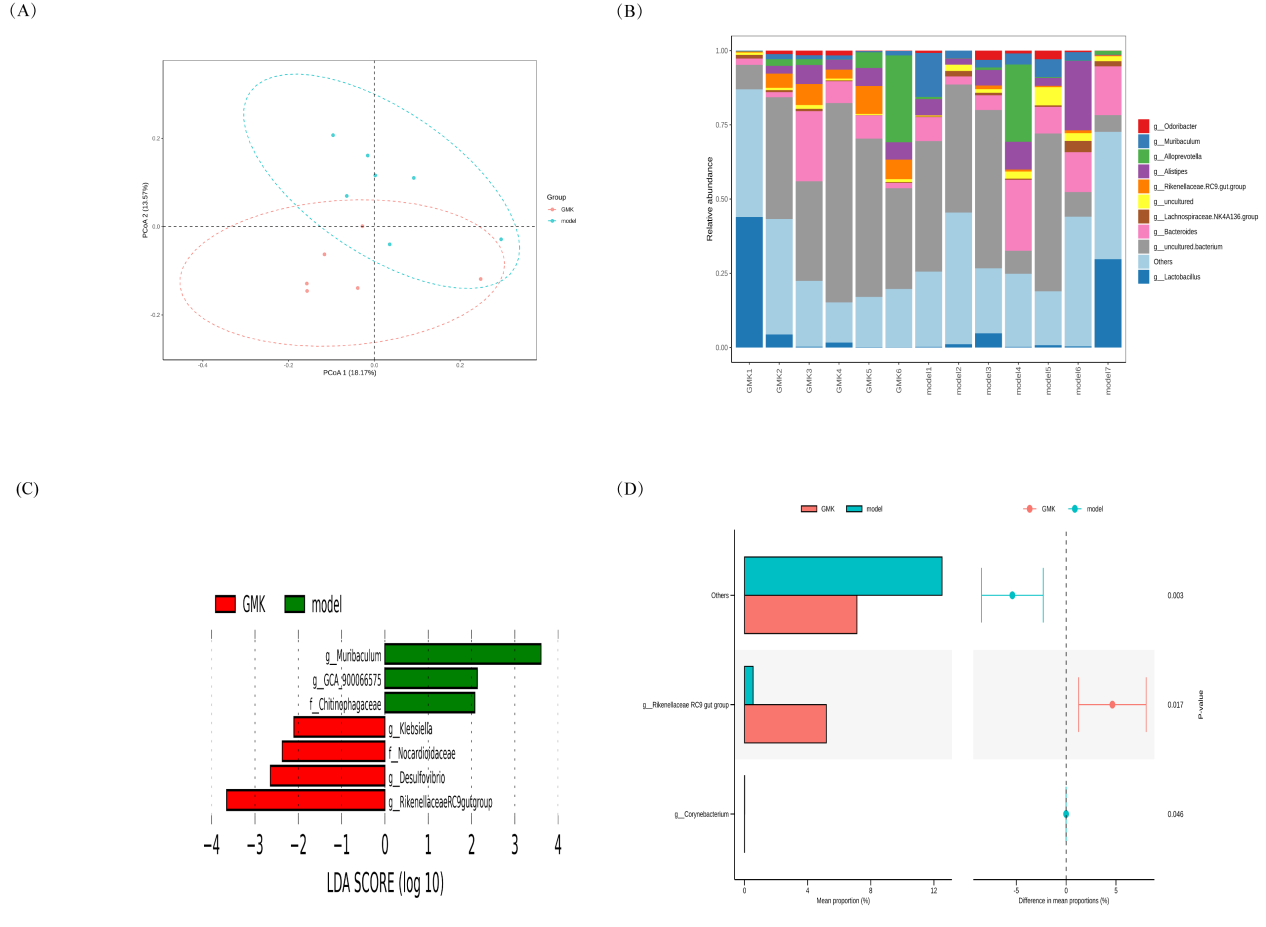
**

**Figure 6 Gut microbiota Biomarker detection after Guominkang treated.** (A) Principal coordinate analysis (PCoA) of gut microbiota in GMK treated group and eosinophilic asthma model group; (B) The top 10 gut microbiota at genus level between GMK treated group and model group; (C) Lefse analysis of differential gut microbiota between GMK treated group and model group (LDA > 2 was used); (D) STAMP analysis of differential gut microbiota between GMK treated group and model group (p < 0.05 was used), n=7/group.

**
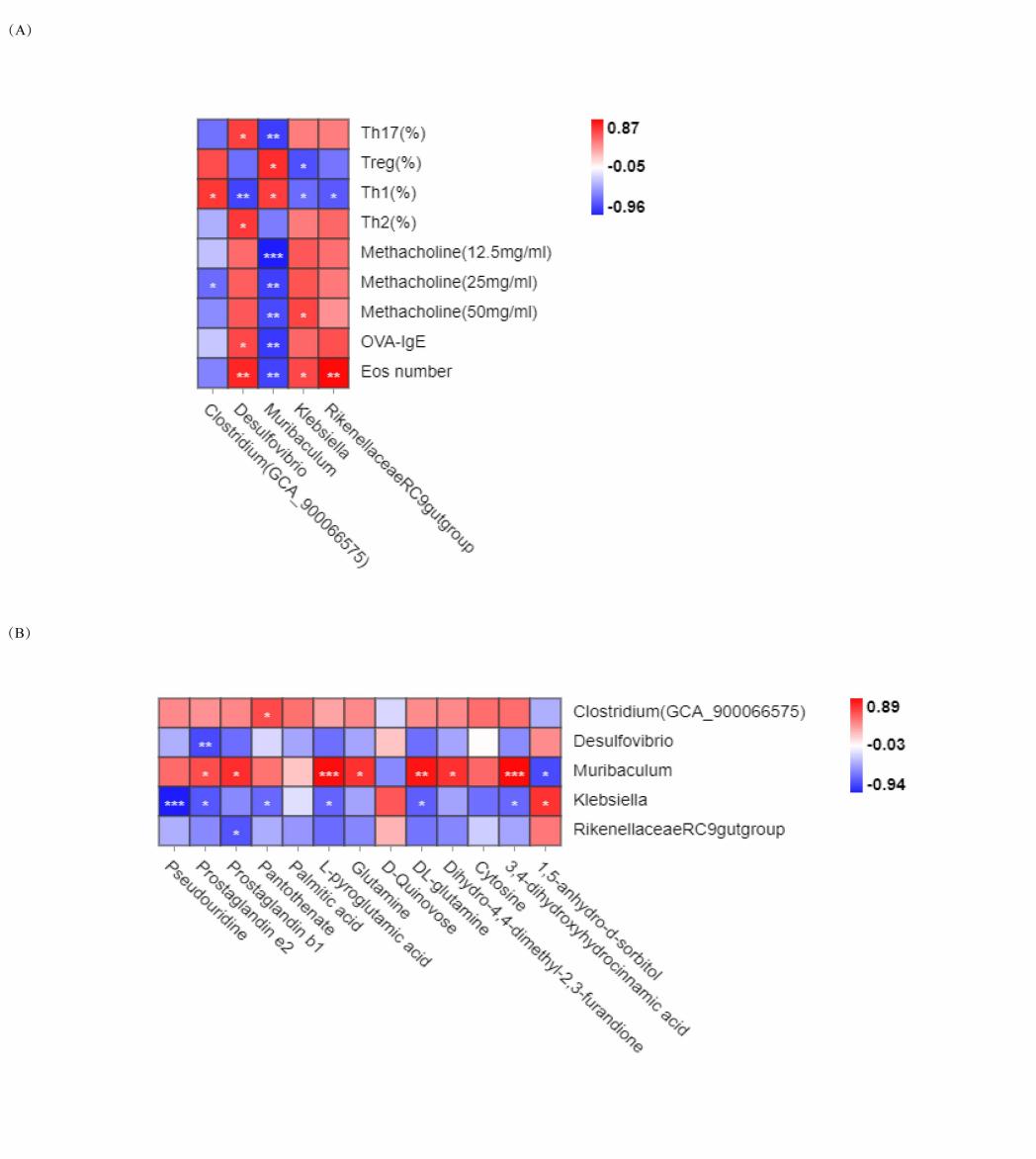
**

**Figure 7 Correlation analysis between gut microbiota and Th1/2, Treg / Th17 cell balance in eosinophilic asthma.** (A) Correlation analysis between differential microbiota and Th1,Th2, Treg and Th17 cell percentage, Penh value, OVA-IgE antibody and eosinophil number in BALF. (B) Correlation analysis of diffenential metabolites and gut microbiota biomarker treated by Guominkang (n=5-6/group). * *p* < 0.05, ***p* < 0.01, *** *p* ≤ 0.001.
