# Supplementary material for: Guominkang formula alleviate inflammation in eosinophilic asthma by regulating immune balance of Th1/2 and Treg/Th17 cells
Source: Front Pharmacol. 2022 Oct 14;13:978421. doi: 10.3389/fphar.2022.978421 (PMC9624229; doi:10.3389/fphar.2022.978421)
Supplement: Supplementary file 9 [file Table10.DOCX]

HE staining:

<https://www.jianguoyun.com/p/DbRLVKQQ7YPbChjFhMkEIAA>

PAS staining:

<https://www.jianguoyun.com/p/DbhG6NgQ7YPbChjHhMkEIAA>

RT-qPCR:

<https://www.jianguoyun.com/p/DdN-P1YQ7YPbChjJhMkEIAA>

Th1,Th2,Treg,Th17:

<https://www.jianguoyun.com/p/DYovSVUQ7YPbChjLhMkEIAA>

16S rDNA raw data: It has also been deposited within NCBI, the correct accession is: PRJNA850513，and the data will released on June 30^th^.
